# 650. Re-evaluating Resuscitation Thresholds in Elderly Burn Patients

**DOI:** 10.1093/jbcr/irag033.487

**Published:** 2026-04-06

**Authors:** Hannah M L Olson, Alexandra M Lacey, Nicholas Larson

**Affiliations:** University of Minnesota, Minneapolis, MN; Regions Hospital, Saint Paul, MN; Regions Hospital Burn Center, Minneapolis, MN

## Abstract

**Introduction:**

Elderly burn patients are at increased risk of adverse outcomes at a lower total body surface area (TBSA) than their younger counterparts due to a higher burden of comorbidities and decreased physiologic reserve. Standard of care is to activate nurse-driven resuscitation protocols (NDRP) for burns involving ≥20% TBSA. After observing a concerning trend of adverse events in patients aged ≥60 with 15-19.9% TBSA burns, our burn center revised its protocol to initiate NDRP in patients with ≥15% TBSA.

**Methods:**

This is a retrospective cohort study of 47 adult burn patients (≥18 years) presenting with 15-19.9% TBSA burns admitted to our burn center between January 2020 through April 2025. Patients were grouped by age ≥ 60 (n = 16) and age < 60 (n = 31). The primary outcomes were mortality and incidence of acute kidney injury (AKI) in the first 48 hours of admission. A Fisher’s exact test was utilized due to the small sample size of this study.

**Results:**

Patients in the age ≥ 60 group experienced significantly higher mortality compared to the age < 60 group (3/16 vs. 0/31 deaths; *p*=.033). Although the incidence of AKI within 48 hours of admission was greater in the age ≥ 60 group (4/16 vs. 1/31), the difference was not statistically significant (*p*=.138) likely due to the small sample size. However, the relative risk of AKI in patients age ≥ 60 was 7.75, indicating a clinically meaningful trend toward increased AKI risk in this population.

**Conclusions:**

Burn patients aged ≥60 years with TBSA 15-19.9% demonstrate higher risk of mortality and a trend towards increased AKI rates compared to their younger counterparts. These results support the adoption of a lower threshold for initiating NDRP to 15% TBSA in patients aged ≥60, extending protocol indications to this vulnerable population.

**Applicability of Research to Practice:**

These results support the adoption of a lower threshold for initiating NDRP to 15% TBSA in patients aged ≥60.

**Funding for the study:**

N/A.

